# Supplementary material for: Endosomal chloride/proton exchangers need inhibitory TMEM9 β-subunits for regulation and prevention of disease-causing overactivity
Source: Nat Commun. 2025 Apr 1;16:3117. doi: 10.1038/s41467-025-58546-3 (PMC11962092; doi:10.1038/s41467-025-58546-3)
Supplement: Supplementary file 1 — Supplementary Information [file 41467_2025_58546_MOESM1_ESM.pdf]

Supplementary Material for Planells-Cases *et al*.

'*Endosomal chloride/proton exchangers need inhibitory TMEM9 β-subunits for regulation and prevention of disease-causing overactivity*'

Supplementary Figures

Supplementary Tables

Supplementary Note

Supplementary References

Supplementary Figures

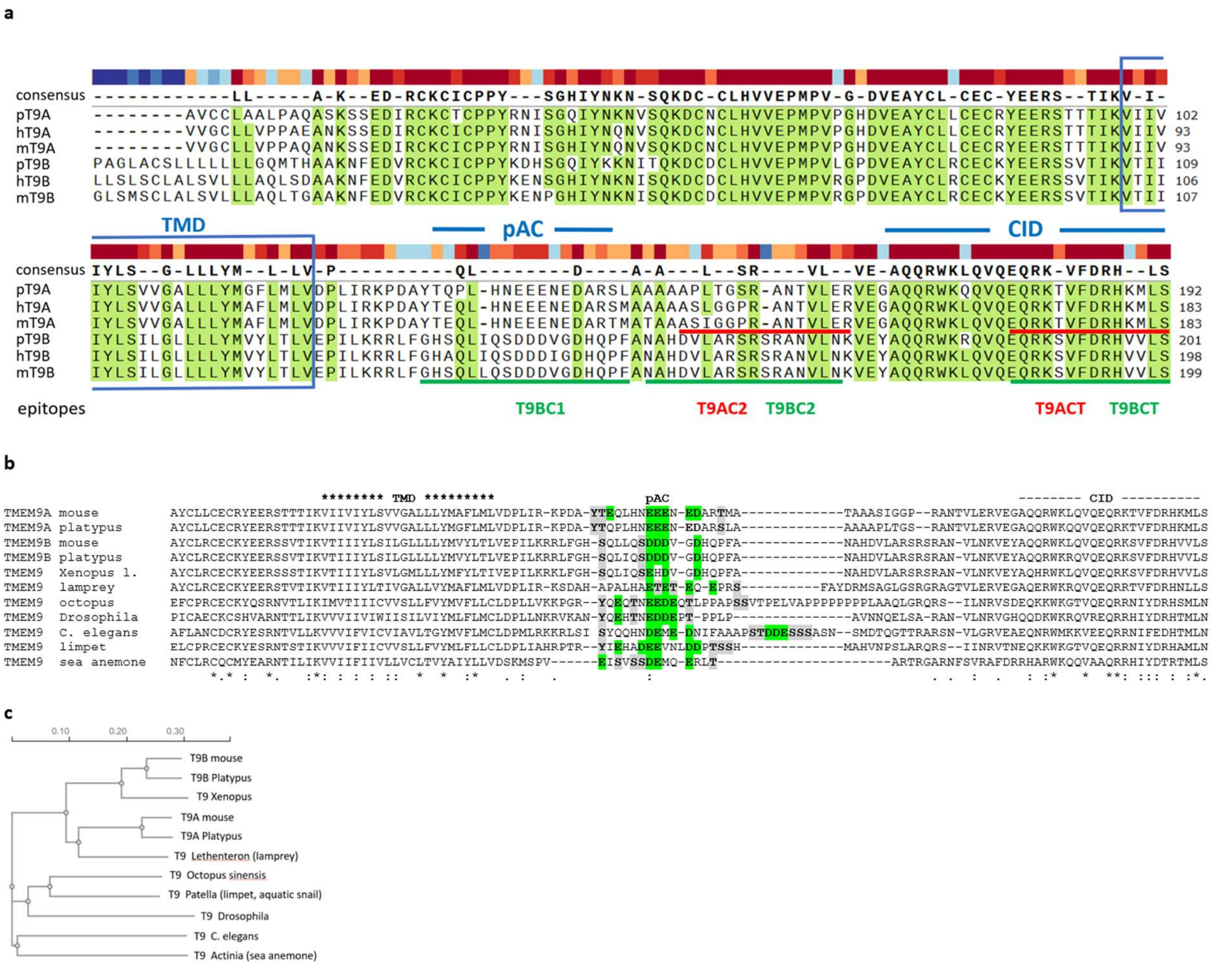

Supplementary Figure 1. **Conservation and features of TMEM9 proteins**. (**a**) Alignment of selected mammalian T9A and T9B proteins, ignoring the extreme signal peptide-containing N-terminus of the precursor protein. Prefix indicates species (p, platypus; h, human; m, mouse). Predicted extent of the transmembrane domain (TMD), the C-terminal inhibitory domain CID, and the epitopes used to generate antibodies used in this work, are indicated. (**b**) Partial alignment of TMEM9 proteins across distant animal species, with emphasis on phosphorylated acidic clusters (pAC). Sequences shown begin 20 residues before the TMD. Alignment obtained by Clustal W. Mammals including the egg-laying platypus have two different isoforms (TMEM9 and TMEM9B). Sequence conservation is highest in the TMD and adjacent regions and in the ~25 last amino acids. Although not conserved at the sequence level, all TMEM9 proteins contain pAC (phosphorylated acidic cluster) motifs about 15 residues after the TMD. This motif is characterized by negatively charged amino-acids (green) and (potentially phosphorylated) serines, threonines or tyrosines (grey). Databases (www.phosphosite.org/) indicated the following number of instances of phosphorylated residues: T9A: ^{137}T 19 times; T9B: ^{138}S 6 times, ^{143}S 5 times). Our proteomic analysis from mouse brain confirmed phosphorylation of T9B ^{138}S and ^{143}S and newly revealed phosphorylation of ^{122}Y and ^{123}T (Supplementary Fig. 6). Note high conservation of the last ~20 amino-acids corresponding to CID determined by our functional assays. (**c**) Dendrogram showing the phylogenetic relationship between the isoforms and orthologs shown in (a,b) obtained by Clustal W. (Accession numbers: sp|Q9CR23|TMEM9_MOUSE; XP_028924314.1 TMEM9 *Ornithorhynchus anatinus* (Platypus); sp|Q9JJR8| TMM9B_MOUSE; XP_028917020.1 TMEM9B *Ornithorhynchus anatinus*;

XP_018115833.1 *Xenopus laevis*; XP_061409818.1 *Lethenteron reissneri* (lamprey); XP_029645748.1 *Octopus sinensis*; AAL90411.1 *Drosophila melanogaster*; NP_001040796.1 *C. elegans*; XP_050393211.1 *Patella vulgate* (limpet); XP_031565082.1 TMEM9 *Actinia tenebrosa* (sea anemone)).

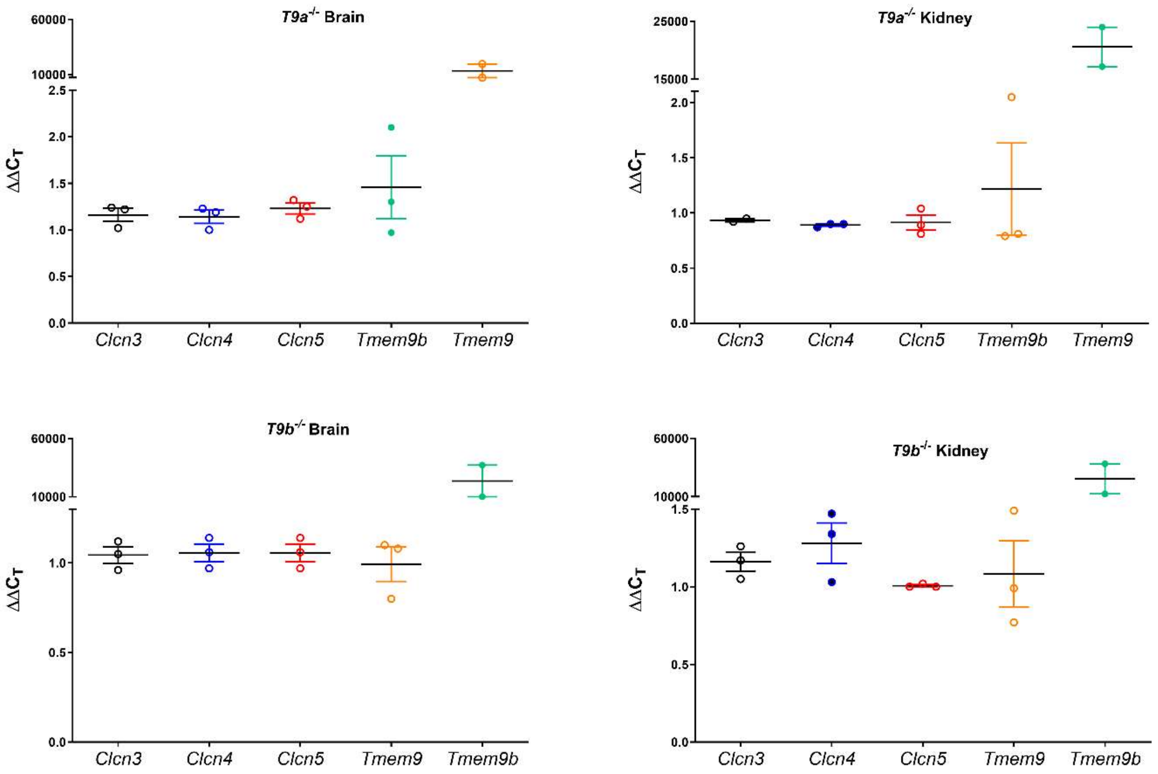

Supplementary Figure 2. **Changes in transcript levels for endosomal CLC exchangers and T9 subunits** in *T9a*$^{-/-}$ and *T9b*$^{-/-}$ adult mouse kidneys and brains compared to respective WT tissues as determined by qRT-PCR. Except for the disrupted genes, no significant changes in levels are seen by qRT-PCR. Data are given as mean ± SEM.

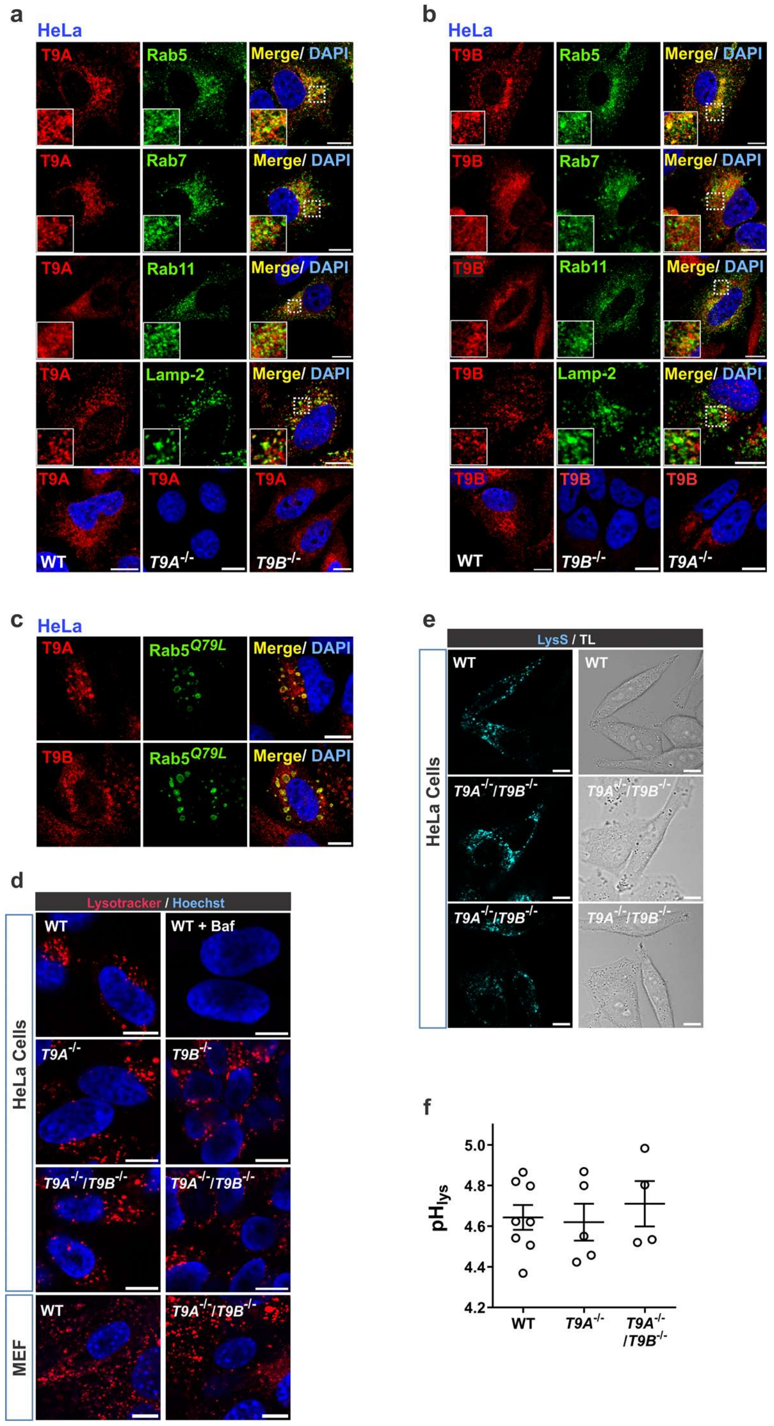

Supplementary Figure 3. Subcellular localization of T9A and T9B in HeLa cells (a-c) and lack of an effect of either TMEM9 on lysosomal pH (pH_{lys}) (d-f). **(a,b)** Co-labeling of endogenous T9A (a) and T9B (b) with rab5, rab7, rab11, and lamp2 in HeLa cells. Native cells, except for rab11, for which rab11 was overexpressed. **(c)** Natively expressed T9A and T9B localize to endosomes enlarged by $rab5^{Q79L}$ transfection. **(d)** No difference in Lysotracker® or **(e)** Lysosensor® staining between WT, *T9A*$^{-/-}$, *T9B*$^{-/-}$ and *T9A*$^{-/-}$/*T9B*$^{-/-}$ HeLa cells (results from two independent clones shown). Inhibition of H^+-ATPase with bafillomycin (Baf) in (d) serves as control. Similar Lysotracker staining was found in WT and *T9a*$^{-/-}$/*T9b*$^{-/-}$ MEFs. Scale bars, 10 µm. **(f)** Ratiometric measurements of lysosomal pH (pH_{lys}) with Oregon Green 488 dextran loaded by endocytosis into lysosomes in WT, *T9A*$^{-/-}$ and *T9A*$^{-/-}$/*T9B*$^{-/-}$ HeLa cells. Each individual data point derived from one dish. For WT n=8, for *T9A*$^{-/-}$ n=5 and for *T9A*$^{-/-}$/*T9B*$^{-/-}$ n=4. Data given as mean ± SEM.

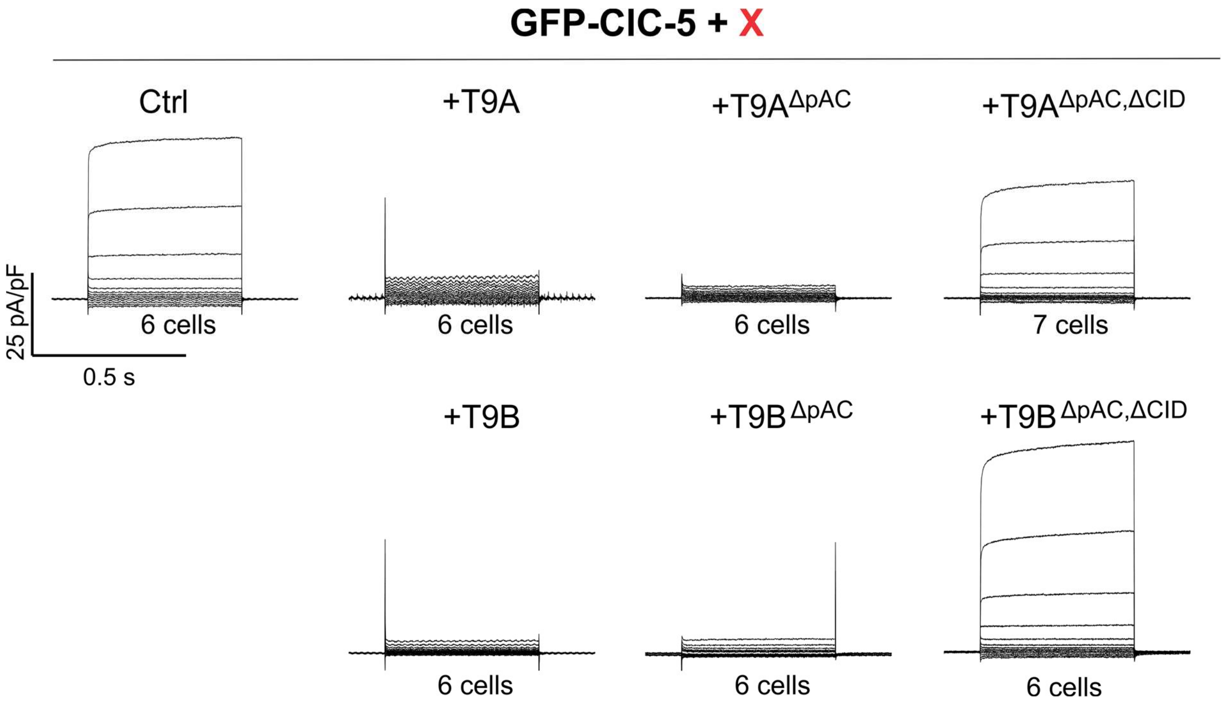

Supplementary Figure 4. **T9A and T9B suppress ClC-5 currents in transfected *T9A*$^{-/-}$/*T9B*$^{-/-}$ HeLa cells.** As in oocytes (Fig. 3), T9A and T9B suppress ClC-5 currents. This suppression is absent with double mutants in pAC and CID motifs which allow surface expression and relieve current inhibition, respectively. *T9A*$^{-/-}$/*T9B*$^{-/-}$ cells, used to avoid background of endogenous T9A and T9B, were transfected with expression plasmids encoding GFP-ClC-5 and the indicated T9 proteins and examined using whole-cell patch clamp. Averaged current densities from the indicated number of cells.

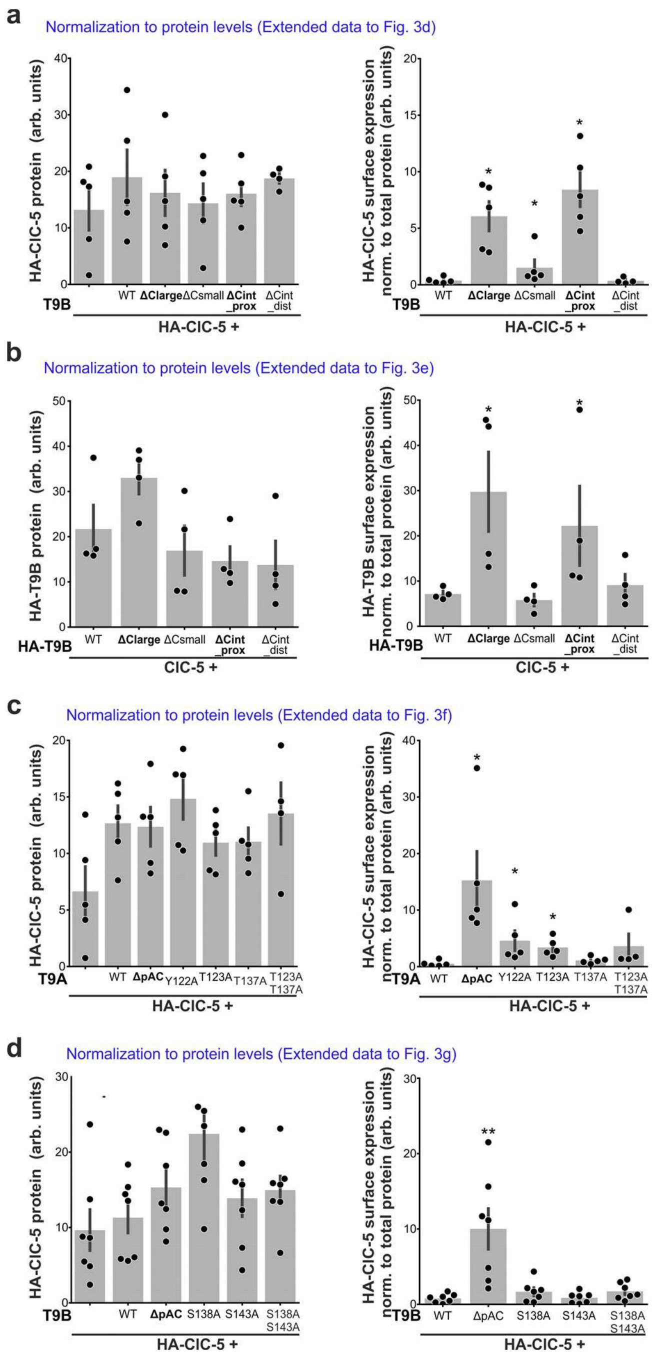

Supplementary Figure 5. Quantification of heterologous protein expression in oocytes for normalization of surface expression data from Fig. 3. **(a)** Protein levels of HA-ClC-5 co-expressed with T9B (WT and mutants) in *Xenopus* oocytes (left) and HA-ClC-5 surface expression normalized to these values (right). **(b)** Protein levels of HA-T9B and its mutants co-expressed with ClC-5 (left), and HA-T9B surface expression normalized to thus obtained levels of total T9B protein (right). **(c,d)** Levels of HA-ClC-5 protein co-expressed with T9A (c) or T9B (d), WT and mutations in pAC (left), and corresponding normalized surface expression of HA-ClC-5 (right). Data shown as mean ± SD. *, $p < 0.05$; **, $p < 0.01$, using non-parametric two-tailed Mann–Whitney test using the Benjamini–Hochberg correction. Numerical data and exact p values are given in Source Data provided with this paper. Data are compared to HA-ClC-5 + T9B (a, d), to ClC-5 + HA-T9B (b) or to HA-ClC-5 + T9A (c).

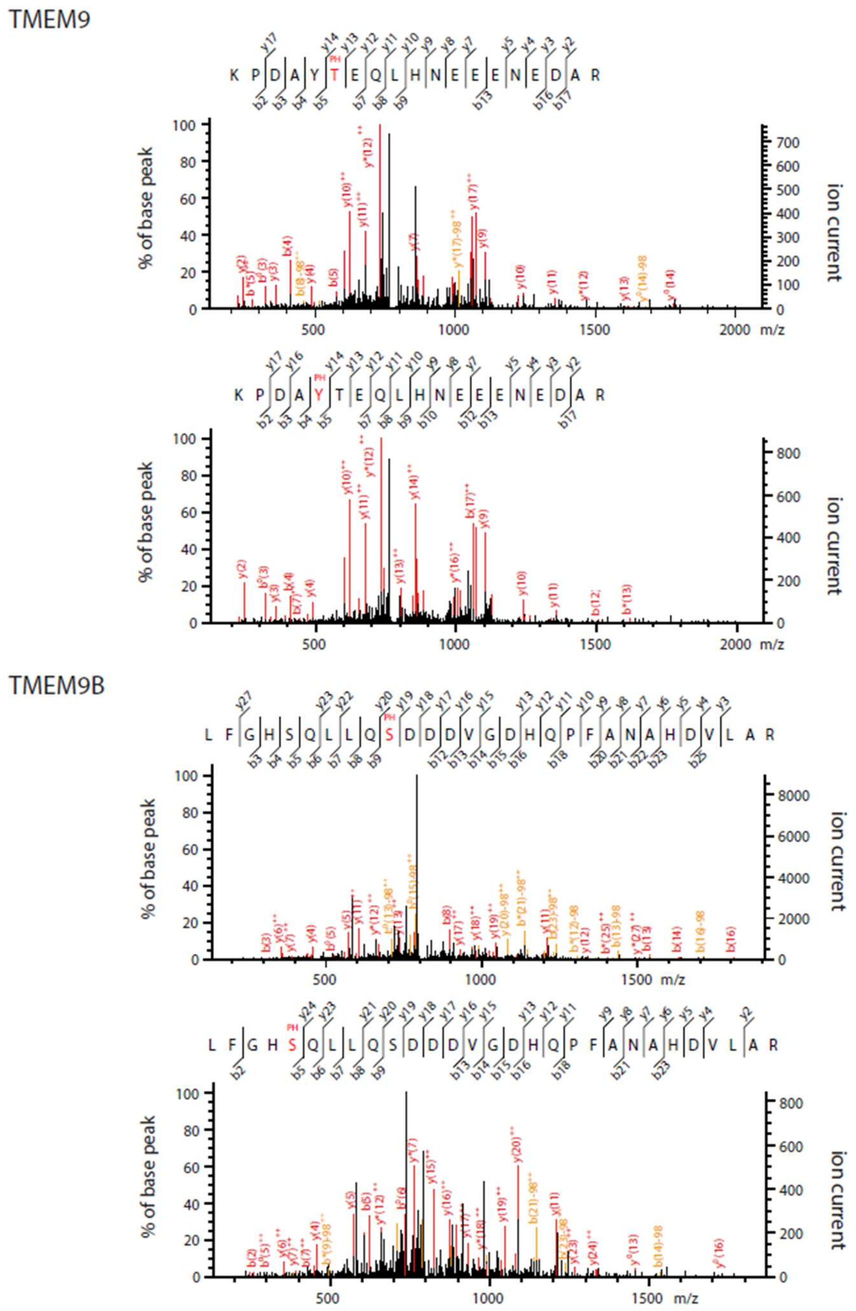

Supplementary Figure 6. Phosphorylated residues in T9A and T9B phospho-spectra. Exemplary MS/MS spectra (of n = 2 - 10) of peptides from TMEM9 and TMEM9B (from AP-MS analyses without phosphor-peptide enrichment, *Methods*) identifying unambiguous phosphorylation sites in the respective proteins. MASCOT-specific quality parameters are indicated, positional specificity was 97% (S) / 99% (S) for TMEM9B and 100% (Y) / 100% (S) for TMEM9 sites.

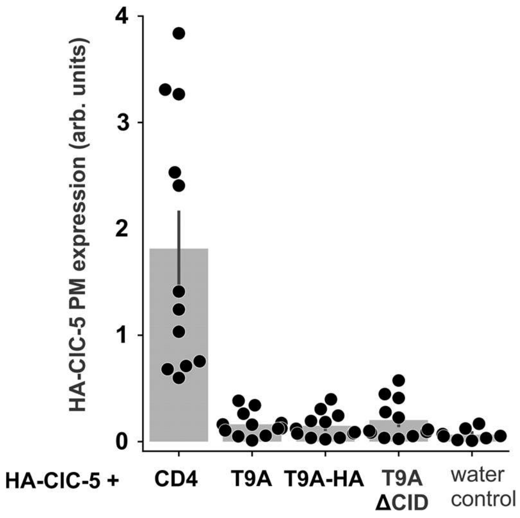

Supplementary Figure 7. Suppression of HA-ClC-5 surface expression by T9A is not affected by the C-terminal addition of an HA epitope or the ΔCID mutation. Co-expression with CD4 or water injection were used as controls. Data represented as mean ± SD as individual oocytes from a single experiment that were confirmed by an additional independent experiment. Numerical data is given in Source Data provided with this paper.

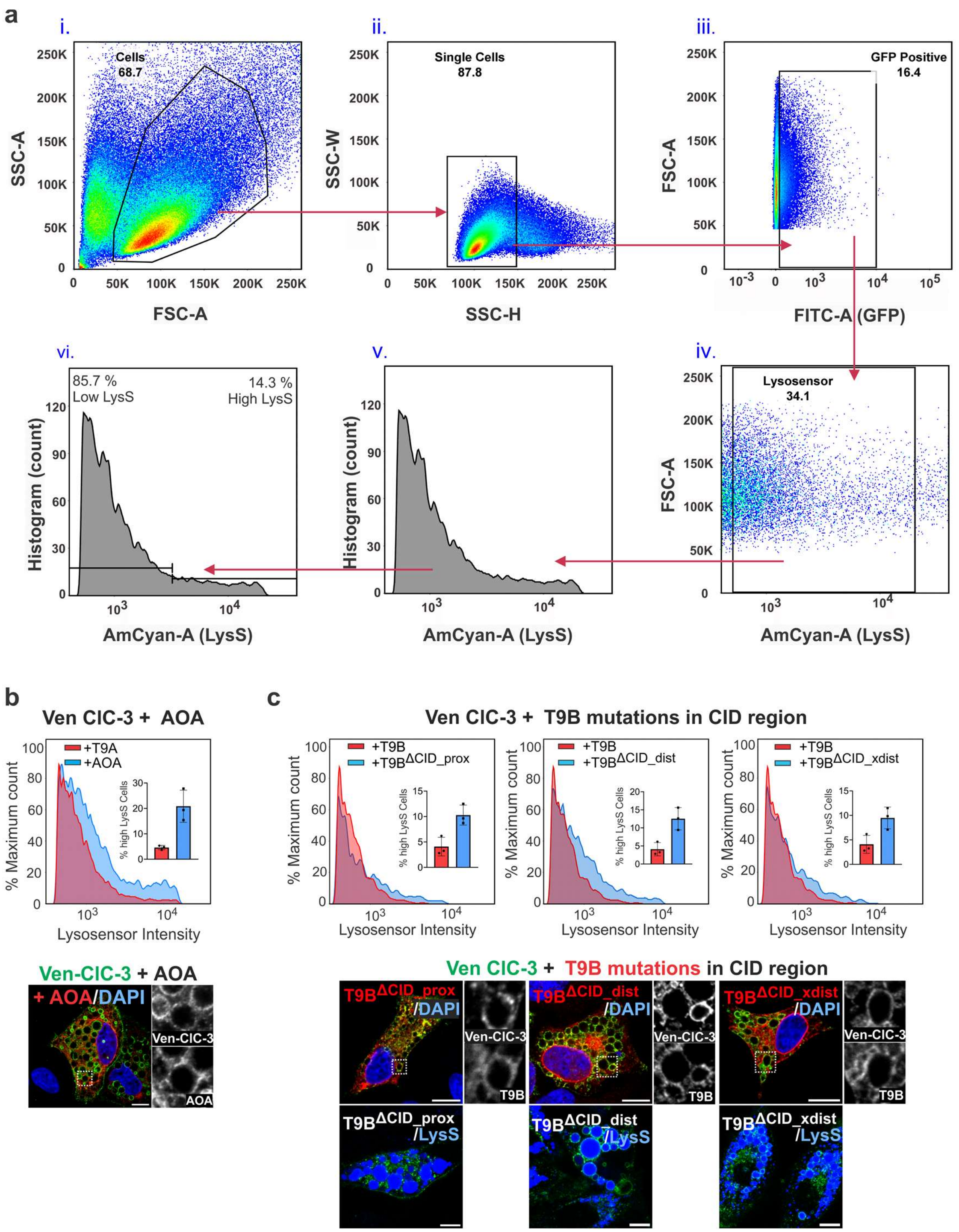

Supplementary Figure 8. Flow cytometry to detect large vacuoles. (a) Gating strategy for the identification of GFP-positive and Lysosensor-positive populations. (i) Cells were initially identified based on forward scatter area (FSC-A) and side scatter area (SSC-A), which represent cell size and granularity, respectively. Events falling within the gated region were classified as cells. (ii) Single-cell events were distinguished from doublets or aggregates by plotting side scatter height (SSC-H) against side scatter width (SSC-W). Single cells were selected for analysis, minimizing the inclusion of multiplets. **(iii)** From the single-cell population, GFP-positive cells were detected based on their fluorescence in the FITC-A channel, indicating expression of the GFP signal. GFP-positive cell events were carried forward for downstream

analysis. (iv) The GFP-positive population was then assessed for acidic vesicle content using LysoSensor. LysoSensor-positive cells were identified based on fluorescence in the AmCyan-A channel. (v) Distribution of LysoSensor-positive cells based on AmCyan-A fluorescence shown as histogram. The y-axis represents the counts of events and highlights the frequency distribution of AmCyan-A fluorescence intensity which reflects variability in lysosomal content within the GFP-positive population. (vi) Through the bisector tool, threshold gating was applied at (mean Lysosensor intensity + 2 x SD) and through the mentioned percentages, high and low intensity Lysosensor containing cells were marked within the population. **(b)** Suppression of vacuolization requires physical ClC-3/T9A association. It is impaired with chimera AOA in which the T9A TMD is replaced by that of Ostm1. FC top, IF bottom. **(c)** Extension of CID determined by functional analysis of alanine scanning mutants. FC top, IF, middle, Lysosensor-filled vacuole staining, bottom. Note that in insets of FC panels for T9B mutants in CID (panel c, top) the percentage of cells displaying 'high Lysosensor intensity' was compared to the same data set of T9B expressing cells. Data values in panel (b, c) represent percentage of cells exhibiting high Lysosensor intensity from three individual biological replicates. Data shown as mean ± SD. Non-parametric two-tailed Mann-Whitney test yielded $p = 0.1$. Numerical data and exact p values given in Source Data. Same data set for ClC-3 + T9B (red, in all insets of panel c) was used. Scale bars, 10 µm.

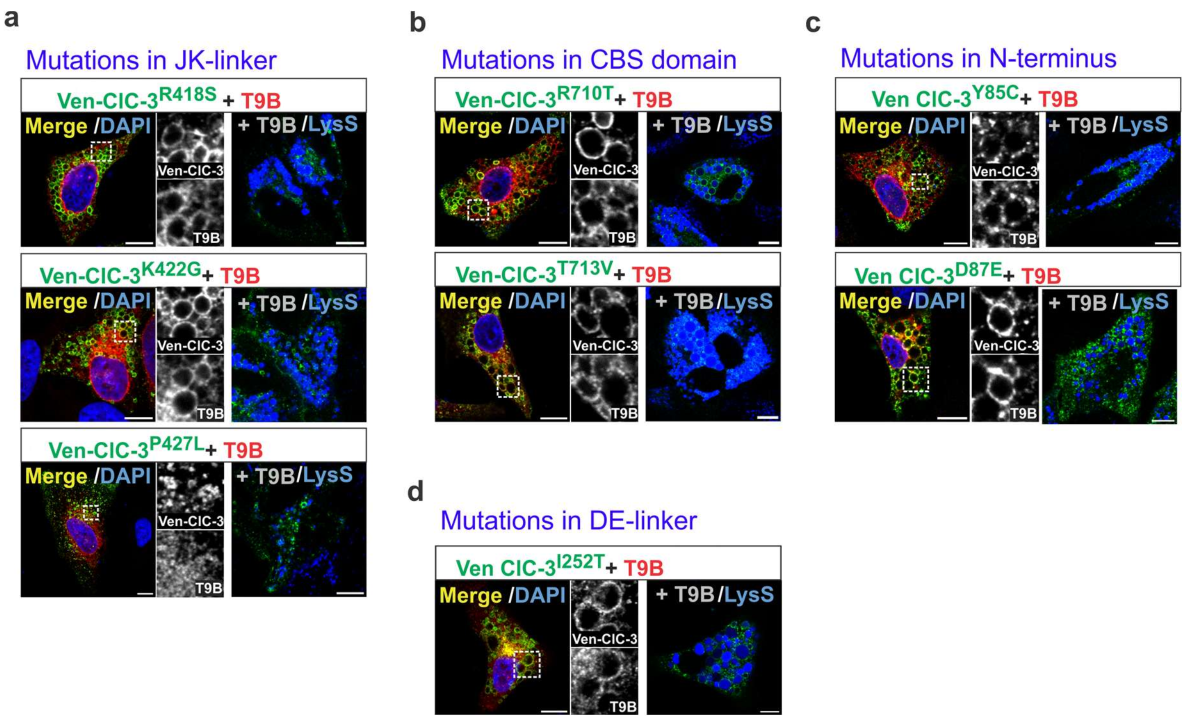

Supplementary Figure 9. Effect of T9B co-expression on effects of pathogenic ClC-4 mutants in JK linker **(a),** CBS1 **(b),** N-terminus **(c)** and DE linker **(d),** all studied in ClC-3 backbone**.** Left, immunocytochemistry with labeling for Venus-ClC-3 (green) and T9B (red); right, live cell imaging showing pH-sensitive Lysosensor® fluorescence (blue). CLC mutants are similarly resistant to T9B as to T9A, the latter shown in main Fig. 5c-g. Scale bars, 10 µm

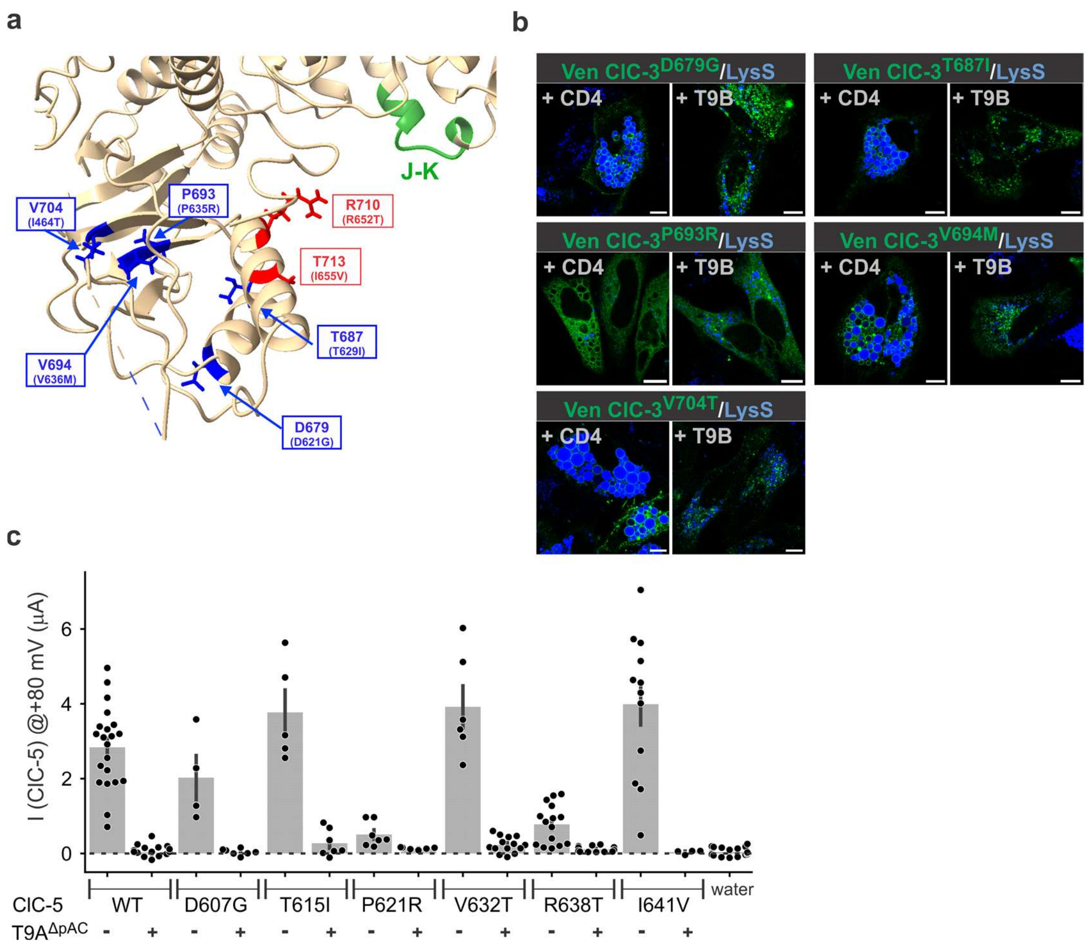

Supplementary Figure 10. **Functional analysis of pathogenic ClC-4 mutants in CBS1** in backbone of ClC-3 and ClC-5. **(a)** Location of pathogenic *CLCN4* mutations in CBS1 (ref.[1]) depicted in the structure of ClC-3. ClC-4 missense variants are shown in brackets below the name of the respective ClC-3 residues. When equivalent mutations were inserted into the ClC-3 cDNA, only R710T and T713V interfered with T9-mediated inhibition of vacuolization (Fig. 5d). These residues (shown in red) are located in the 2nd α-helix of CBS1 and face the cytoplasm where they might interact with CIDs of T9 subunits. The stretch of the J-K linker which was replaced by the equivalent sequence of ClC-2 in $T9^{JK-C2}$ mutants (^{416}RRRKST to QVMRKQ, which robustly interfered with CID inhibition (Fig. 5a, b)) is shown in green. Our data suggest that CID interacts, directly or indirectly, with R710, T713 and the JK linker. Note that the residues mutated in other pathogenic CBS1 variants of ClC-4 rather point to the interior of CBS1 where they are unlikely to contact CID. Their mutation did not relieve suppression of vacuolization in our functional assay and provide an important negative control. (Note that the N-terminus of the 'long' ClC-3 splice variant ClC-3b, which is commonly used to number ClC-3 residues, is extended by 58 residues compared to ClC-3a, which was used in our work, and to ClC-4 and ClC-5, resulting in different numbering of equivalent residues). **(b)** Functional vacuolization assay for Ven-ClC-3 T687I, D679G, V694M, V704T, P693R mutants. No relief from T9B blockage is observed, in contrast to R710T and T713V (Fig. 5d). Scale bars, 10 µm. **(c)** Currents of ClC-5 mutants modeled on pathogenic *CLCN4* CBS1 mutants[1]. Currents from all mutants were suppressed by $T9A^{\Delta pAC}$ which allows surface expression, but retains CID intact. Suppression was also observed with R638T and I641V, although the equivalent mutants R710T and T713V in ClC-3 were partially T9-resistant in the more sensitive vesicle assay (Fig. 5d,e, Supplementary Fig. 9b). Data shown as mean ± SD, with numerical data given in Source Data provided with this paper.

Supplementary Tables

SUPPLEMENTARY TABLE 1

Modified protein sequences (red) of HA-tagged versions of T9A, T9B and ClC-5.

Protein	Amino acid sequence of HA-epitope with flanking linkers
HA-T9B	[27]LLAQLTGAAK*VREGGSYPYDVPDYAGYPYDVPDYAGSYPYDVPDYASK*NFEDVRCK
T9B-HA	[189]KSVFDRHVVLS*LVPGYPYDVPDYAGYPYDVPDYAGSYPYDVPDYAV**
T9A-HA	[173]KTVFDRHKMLS*LVPGYPYDVPDYAGYPYDVPDYAGSYPYDVPDYAV**
HA-ClC5	[408]NTSKGGELPD*SGREPTTPGSGSSTTPTPGPTTGGSSPTSTPNGEPTTPGSGSSTTPTPGPTTGGSSP TLINLYPYDVPDYASPTPNGDYPYDVPDYASPTTPGYPYDVPDYALINSGSSTTPTPGPTTGDSSPTST PNGELSPTTPGSGSSTTPTPGPTTGGSSPTSTPNGGG*RPAGVGVYSA

SUPPLEMENTARY TABLE 2. List of antibodies used and attribution to figures

Antibody	Sp	Use (dil.)	Company	Article Nr./ Ref.	Used in Figure
ClC-3	Rb	WB (1:500)	C.-M. (Eurogentec)	Stobrawa et al., 2001	Fig. 2b-f
ClC-4	Rb	WB (1:500)	C.-M. (Eurogentec)	N/A	Fig. 2b-f
ClC-5	gp	IHC (1:200)	C.-M. (Eurogentec)	N/A	Fig. 2g, upper panel
ClC-5	Rb	IF (1:250)	C.-M. (Eurogentec)	N/A	Fig. 2j
ClC-5	Rb	IHC (1:250)	C.-M. (Eurogentec)	Piwon et al., 2000	Fig. 2g, lower panel
ClC-7	Rb	IHC (1:100)	C.-M.	Kornak et al., 2001	Fig. 2i
ClC-7	Rb	WB (1:500)	C.-M.	Kornak et al.,2001	Fig. 2b-c
GAPDH	Ms	WB (1:2000)	Sigma Aldrich	G8795	Fig. 2a
GFP	Chk	IF (1:500)	Aves Lab	GFP-1020	Fig. 3j, 4a, 4d-h, 5c-d, 5f-g, 6a-d, Suppl. fig. 8b-c, Suppl. fig. 9
GFP	Ms	WB (1:1000)	Sigma Aldrich	T5168	Fig. 1d
GFP	Ms	IP	Roche	11814460001	Fig. 1b, 1c
GFP	Gt	IP	R&D Systems	AF4240	Fig. 1b, 1c
HA	Rat	IF (1:250)<br>PM (1:1000)	Roche	11867423001	Fig. 3d-g<br>Fig. 4f-h
HA	Rb	WB (1:1000)	Cell Signaling	3724	Fig. 1c
Lamp1	Rat	IHC (1:200)	BD Pharmingen	553792	Fig. 2i
Lamp1	Rb	WB (1:500)	Abcam	ab24170	Fig. 2c
Lamp2	Ms	IF (1:500)	Abcam	Ab25631	Suppl. fig. 3a-b, Fig. 4a, 4d, Fig. 5c-d, Fig. 5f-g, Fig. 6a, 6c
Rab5	Ms	WB (1:1000)	Synaptic Systems	108011	Fig. 2c, Suppl. fig. 3c
Rab5	Rb	IF (1:1000)	Abcam	ab218624	Suppl. fig. 3a-b
Rab7	Rb	WB (1:1000)	Abcam	ab137029	Suppl. fig. 3a-b
Rab11	Rb	IF (1:250)	Zymed/ Invitrogen	71-5300	Suppl. fig. 3a-b
TfR	Ms	WB (1:500)<br>IF (1:500)	Thermo Fisher Scientific	13-6800	Fig. 2c
TMEM9 "T9AC2"	Rb 1	WB (1:1000)<br>IP	C.-M. (Pineda, Berlin)	N/A	Fig. 1b, 2c
TMEM9 "T9AC2"	Rb 2	IP	C.-M. (Pineda, Berlin)	N/A	Fig. 1b
TMEM9 "T9AC2"	GP	WB (1:1000)<br>IF (1:2000)	C.-M. (Pineda, Berlin)	N/A	Fig. 2a-b, Fig. 2d-f, Fig. 3j, Fig. 4d-h, Fig. 5c-d, Fig. 5f-g, Fig. 6a, Fig. 6c-d, Suppl. fig. 8c
TMEM9 "T9ACt"	Gp1	IF (1:200)	C.-M. (Eurogentec)	N/A	Suppl. fig. 3a, 3c
TMEM9 "T9ACt"	Rb1	IHC (1:1000)	C.-M. (Eurogentec)	N/A	Fig. 2g-h
TMEM9 "T9ACt"	Rb2	IP	C.-M. (Eurogentec)	N/A	Fig. 1b
TMEM9B	Rb	IP	Proteintech	24331-1-AP	Fig. 1c
TMEM9B "T9BC1"	Rb 2	IP	C.-M. (Pineda, Berlin)	N/A	Fig. 1c
TMEM9B "T9BC2"	Rb 1	WB (1:5000)<br>IP<br>IF (1:1000)	C.-M. (Pineda, Berlin)	N/A	Fig. 1b, Fig. 2a-f, Fig. 4d-e, Fig. 6a-d, Suppl. fig. 8c, Suppl. fig. 9
TMEM9B "T9BCt"	Gp 2	IHC (1:800)<br>IF (1:800)	C.-M. (Pineda, Berlin)	N/A	Fig. 2g-j<br>Suppl. fig. 3b-c

Chk=Chicken, C.-M.=Custom-made; Gp=guinea pig; Gt=Goat; IF= Immunofluorescence; IHC=Immunohistochemistry; PM= surface expression; IP=Immunoprecipitation; Ms=mouse; Rb=Rabbit; WB= Western blot; N/A= No applied; SP=Host specie

Supplementary Note

Endolysosomal $2Cl^-/H^+$-exchange and vacuole formation

Many of the giant vesicles observed with excessive CLC antiport activity were poorly acidified. This argues against H^+-gradient driven Cl^- accumulation, although the round shape of vesicles suggested increased luminal pressure. This apparent contradiction is resolved by noting that $2Cl^-/H^+$-exchangers transport three negative charges per transport cycle. Thermodynamics (see below) shows that an inside-positive potential of +25 mV suffices to accumulate Cl^- to 130 mM. The positive voltage would also inhibit vesicular CLCs and many lysosomal ion channels. We propose that these poorly acidified vesicles have reached a quiescent steady-state. This contrasts with acidified vesicles, whose V_{lum} may be around -25 mV and thus allows ion transport by many endolysosomal channels and transporters.

Thermodynamic considerations

It is instructive to consider thermodynamic constraints and predictions for vesicular $2Cl^-/H^+$ exchangers. Their reversal (equilibrium) potential is given by:

$$E_{rev} = (RT/(3F)) * \ln (([Cl^-]_{lum}^2 * [H^+]_{cyt}) / ([Cl^-]_{cyt}^2 * [H^+]_{lum}))$$

where R,T, and F have the usual meaning, E_{rev} compares lumen to cytosol, $[Cl^-]_{lum}$ and $[Cl^-]_{cyt}$ are the luminal and cytosolic Cl^- concentrations, and $[H^+]_{lum}$ and $[H^+]_{cyt}$ the luminal and cytosolic H^+ concentrations, respectively. Chloride concentration is squared because 2 Cl^- ions are transported per cycle. The first term has 3 in the denominator because three charges are transported per cycle.

While $[H^+]_{lum}$ (pH_{lum}) of endosomes and lysosomes is quite well known, and we have reasonable estimates for $[Cl^-]_{lum}$, values, endolysosomal voltages are rather uncertain, with a wide range of lumen-negative and positive voltages being reported[2,3]. We will therefore calculate E_{rev} for 'typical' lysosomes and endosomes to predict voltages at which there will be an influx or efflux of Cl^-. We always take $[Cl^-]_{cyt}$ = 30 mM as typical for non-neuronal cells, and pH_{cyt} = 7.2 ($[H^+]_{cyt} = 6.3*10^{-8}$ M).

For **lysosomes**, we assume pH_{lum} = 4.5 and $[Cl^-]_{lum}$ = 130 mM. The latter value is based on our own unpublished results and results from the Krishnan laboratory[4]. It is also close to the maximum achievable Cl^- concentration because of osmotic constraints.

Using these values, we predict

E_{rev} (CLC, lyso) **= - 25 mV** (lumen negative).

Taking a larger Cl^- gradient ($[Cl^-]_{lum}$ = 140 mM, $[Cl^-]_{cyt}$ = 20 mM) we arrive at **E_{rev} = - 33 mV** (lumen negative).

Lysosomal voltages more positive than E_{rev} will lead to Cl^- uptake through CLCs.

For **endosomes**, we take the values determined by Verkman and colleagues[5] for transferrin-labeled endosomes: ($[Cl^-]_{lum}$ = 46 mM, pH_{lum} = 6.1), again with $[Cl^-]_{cyt}$ = 30 mM and pH_{cyt} = 7.2, and get:

E_{rev} (CLC, endo) = **-29 mV** (lumen negative).

Both reversal potentials are in the same range; they are also at the threshold where all vesicular CLCs (except ClC-6) show steep voltage-dependent activation (with increasing lumen-negative potentials). Intriguingly, also the acid-activated anion channel ASOR/TMEM206[6], as well as the lysosomal cation channels TPC1[7], TRPML1[8] and TRPML2[9] activate in this voltage range. Together with our previous reductionist model calculations for vesicle acidification by a proton pump and parallel CLC exchanger, which predicted V_{lum} = -25 mV[10], and model calculations for macropinosomes which again predicted voltages around – 25 mV[11], we used these voltage-dependences as argument that lysosomal voltages should be -20 to -30 mV (inside-negative)[12].

We therefore **propose that CLC $2Cl^-/H^+$-transport is close to thermodynamic equilibrium along the endocytic pathway** and could even (if not too much inhibited) **contribute to the resting voltages** of these compartments. However, the fact that lysosomes swell in a ClC-7-dependent manner upon PI(3,5)P2 depletion[13] and that activation/disinhibition of all other vesicular CLCs lead to large vesicles suggests that native endolysosomal potentials must be somewhat more positive than the CLC equilibrium potential. This might be achieved by the H^+-ATPase.

The observation of large, **non-acidified vesicles** indicates that they cannot use the pH gradient for luminal Cl^- accumulation. We therefore calculated the potential E_{rev} that would be required to reach $[Cl^-]_{lum}$ = 130 mM with $[Cl^-]_{cyt}$ = 30 mM and equal pH at both sides:

$\mathbf{E_{rev}}$ (CLC, eq pH) = **+ 25 mV** (lumen-positive)

which does not seem outrageous. At this potential, the activity of most known endolysosomal ion transporter and channels, including the CLCs, would be largely inhibited. This inhibition may limit the activity of the H^+-ATPase because of only small neutralizing countercurrents. Small countercurrents would also lead to a more inside positive potential in a positive feed-back loop. We envisage these vesicles to be in a different, rather quiescent metabolic state compared to active endolysosomes.

Considerations for the vesicle enlargement assay

The only partial acidification of large vacuoles might affect the reliability of our Lysosensor® FC assay if acidification varies between constructs and conditions. However, we did not observe a systematic difference in acidification of large vesicles between constructs that gave similar number and size of enlarged vesicles. Moreover, all Lysosensor® FC assays, which have the invaluable advantage of being unbiased and involving a very large number of cells, were accompanied by examining at least two coverslips per condition with confocal microscopy.

A further point to consider is the non-linearity of the vesicle enlargement which proceeds over time, saturating at a point when there is virtually no space left in the cytoplasm. This often leads to cell death. Hence the detection of moderate differences in rates of vesicle generation/enlargements requires a judicious choice of the time elapsing between transfection and analysis. Whereas qualitative conclusions are very safe, since we almost never observed large vesicles when co-transfecting GFP-ClC-3 with either T9A or T9B, comparing the extent of disinhibition by point mutation is only semiquantitative.

Supplementary References

1 Palmer, E. E. *et al.* Functional and clinical studies reveal pathophysiological complexity of *CLCN4*-related neurodevelopmental condition. *Molecular psychiatry* **28**, 668-697 (2023). https://doi.org:10.1038/s41380-022-01852-9

2 Steinberg, B. E., Touret, N., Vargas-Caballero, M. & Grinstein, S. In situ measurement of the electrical potential across the phagosomal membrane using FRET and its contribution to the proton-motive force. *Proc Natl Acad Sci U S A* **104**, 9523-9528 (2007). https://doi.org:10.1073/pnas.0700783104

3 Cang, C. *et al.* mTOR regulates lysosomal ATP-sensitive two-pore Na^+ channels to adapt to metabolic state. *Cell* **152**, 778-790 (2013). https://doi.org:10.1016/j.cell.2013.01.023

4 Chakraborty, K., Leung, K. & Krishnan, Y. High lumenal chloride in the lysosome is critical for lysosome function. *eLife* **6**, pii: e28862 (2017). https://doi.org:10.7554/eLife.28862

5 Hara-Chikuma, M. *et al.* ClC-3 chloride channels facilitate endosomal acidification and chloride accumulation. *J Biol Chem* **280**, 1241-1247 (2005). https://doi.org:10.1074/jbc.M407030200

6 Bae, H. R. & Verkman, A. S. Protein kinase A regulates chloride conductance in endocytic vesicles from proximal tubule. *Nature* **348**, 637-639 (1990). https://doi.org:10.1038/348637a0

7 Cang, C., Bekele, B. & Ren, D. The voltage-gated sodium channel TPC1 confers endolysosomal excitability. *Nature chemical biology* **10**, 463-469 (2014). https://doi.org:10.1038/nchembio.1522

8 Dong, X. P. *et al.* The type IV mucolipidosis-associated protein TRPML1 is an endolysosomal iron release channel. *Nature* **455**, 992-996 (2008). https://doi.org:10.1038/nature07311

9 Chen, C. C. *et al.* TRPML2 is an osmo/mechanosensitive cation channel in endolysosomal organelles. *Science advances* **6** (2020). https://doi.org:10.1126/sciadv.abb5064

10 Weinert, S. *et al.* Lysosomal pathology and osteopetrosis upon loss of H^+-driven lysosomal Cl^- accumulation. *Science* **328**, 1401-1403 (2010). https://doi.org:10.1126/science.1188072

11 Zeziulia, M., Blin, S., Schmitt, F. W., Lehmann, M. & Jentsch, T. J. Proton-gated anion transport governs macropinosome shrinkage. *Nat Cell Biol* **24**, 885-895 (2022). https://doi.org:10.1038/s41556-022-00912-0

12 Polovitskaya, M. M. *et al.* Gain-of-function variants in *CLCN7* cause hypopigmentation and lysosomal storage disease. *J Biol Chem* **300**, 107437 (2024). https://doi.org:10.1016/j.jbc.2024.107437

13 Leray, X. *et al.* Tonic inhibition of the chloride/proton antiporter ClC-7 by PI(3,5)P2 is crucial for lysosomal pH maintenance. *eLife* **11** (2022). https://doi.org:10.7554/eLife.74136
