# Supplementary material for: Endosomal chloride/proton exchangers need inhibitory TMEM9 β-subunits for regulation and prevention of disease-causing overactivity
Source: Nat Commun. 2025 Apr 1;16:3117. doi: 10.1038/s41467-025-58546-3 (PMC11962092; doi:10.1038/s41467-025-58546-3)
Supplement: Supplementary file 2 — Reporting Summary [file 41467_2025_58546_MOESM2_ESM.pdf]

nature portfolio

Corresponding author(s): Prof. Thomas J. Jentsch

Last updated by author(s): 2025/03/02

# Reporting Summary

Nature Portfolio wishes to improve the reproducibility of the work that we publish. This form provides structure for consistency and transparency in reporting. For further information on Nature Portfolio policies, see our Editorial Policies and the Editorial Policy Checklist.

## Statistics

For all statistical analyses, confirm that the following items are present in the figure legend, table legend, main text, or Methods section.

| n/a | Confirmed | |
|---|---|---|
| ☐ | ☒ | The exact sample size (*n*) for each experimental group/condition, given as a discrete number and unit of measurement |
| ☐ | ☒ | A statement on whether measurements were taken from distinct samples or whether the same sample was measured repeatedly |
| ☐ | ☒ | The statistical test(s) used AND whether they are one- or two-sided<br>*Only common tests should be described solely by name; describe more complex techniques in the Methods section.* |
| ☒ | ☐ | A description of all covariates tested |
| ☐ | ☒ | A description of any assumptions or corrections, such as tests of normality and adjustment for multiple comparisons |
| ☐ | ☒ | A full description of the statistical parameters including central tendency (e.g. means) or other basic estimates (e.g. regression coefficient) AND variation (e.g. standard deviation) or associated estimates of uncertainty (e.g. confidence intervals) |
| ☒ | ☐ | For null hypothesis testing, the test statistic (e.g. *F*, *t*, *r*) with confidence intervals, effect sizes, degrees of freedom and *P* value noted<br>*Give P values as exact values whenever suitable.* |
| ☒ | ☐ | For Bayesian analysis, information on the choice of priors and Markov chain Monte Carlo settings |
| ☒ | ☐ | For hierarchical and complex designs, identification of the appropriate level for tests and full reporting of outcomes |
| ☒ | ☐ | Estimates of effect sizes (e.g. Cohen's *d*, Pearson's *r*), indicating how they were calculated |

*Our web collection on statistics for biologists contains articles on many of the points above.*

## Software and code

Policy information about availability of computer code

| | |
|---|---|
| Data collection | Sliced complexome brain fractions were analyzed on a Q-Exactive mass spectrometer coupled to an Ultimate 3000 RSLC nano HPLC system (Thermo Scientific).<br>For immunohistochemistry, immunocytochemistry and live imaging (using Lysotracker and Lysosensor Yellow/Blue), images were acquired with a Zeiss LSM880 confocal microscope using Zen Blue software.<br>For lysosomal pH analysis a Nikon Ti Eclipse widefield Epi-TIRF microscope was used.<br>Electrophysiological currents in frog oocytes were recorded in Turbo-Tec 03 X voltage clamp amplifier (NPI Electronics) and pClamp10 software (Molecular devices). For mammalian cell recordings, a MultiClamp 700B patch clamp amplifier/Digidata 1550B digitizer and pClamp10 software were used.<br>Flow Cytometry data acquisition was performed in LSRFortessa (BD Biosciences). Data were analyzed in FlowJo™ software (v10.6.1). |
| Data analysis | MassAll microscopy images were analysed using FIJI2.0/2.1 version.<br>Electrophysiological recordings were analyzed using the Pandas 2.2.2 and SciPy 1.13.1 libraries for Python 3.12 programming language (Python Software Foundation).<br>For Lysosomal pH measurements, images were analyzed with FIJI image J and a script ComDet v.0.5.5 for spot detection intensity ratio. Values were first assembled and later plotted and statistically analyzed with GraphPad Prism 7.03.<br>For flow cytometry data analysis, primary gating strategy was performed in FlowJo™ software (v10.6.1) and then those values were exported into GraphPad Prism (v10.3.1) for statistical analysis and plotting. |

For manuscripts utilizing custom algorithms or software that are central to the research but not yet described in published literature, software must be made available to editors and reviewers. We strongly encourage code deposition in a community repository (e.g. GitHub). See the Nature Portfolio guidelines for submitting code & software for further information.

## Data

Policy information about availability of data

All manuscripts must include a data availability statement. This statement should provide the following information, where applicable:

- Accession codes, unique identifiers, or web links for publicly available datasets
- A description of any restrictions on data availability
- For clinical datasets or third party data, please ensure that the statement adheres to our policy

Source data are provided with this paper. All other data supporting the findings of this study are available from the corresponding author on reasonable request. Requests for materials should be sent to TJJ. The mass spectroscopy data have been submitted to the appropriate database and accession code is provided in the manuscript.

## Research involving human participants, their data, or biological material

Policy information about studies with human participants or human data. See also policy information about sex, gender (identity/presentation), and sexual orientation and race, ethnicity and racism.

| | |
|---|---|
| Reporting on sex and gender | N/A |
| Reporting on race, ethnicity, or other socially relevant groupings | N/A |
| Population characteristics | N/A |
| Recruitment | N/A |
| Ethics oversight | N/A |

Note that full information on the approval of the study protocol must also be provided in the manuscript.

# Field-specific reporting

Please select the one below that is the best fit for your research. If you are not sure, read the appropriate sections before making your selection.

[x] Life sciences [ ] Behavioural & social sciences [ ] Ecological, evolutionary & environmental sciences

For a reference copy of the document with all sections, see nature.com/documents/nr-reporting-summary-flat.pdf

# Life sciences study design

All studies must disclose on these points even when the disclosure is negative.

| | |
|---|---|
| Sample size | Sample sizes were not predetermined with statistical means but based on standard numbers in the field. Each experiment was performed on at least triplicates. For western blot at least three animals per genotype were investigated. expression |
| Data exclusions | No relevant data excluded |
| Replication | All experiments were repeated independently at least three times. |
| Randomization | No randomization was done |
| Blinding | No blinding was performed, but fields randomly chosen. Several fields were imaged per preparation and by different researchers to avoid acquisition bias. |

# Behavioural & social sciences study design

All studies must disclose on these points even when the disclosure is negative.

| | |
|---|---|
| Study description | *Briefly describe the study type including whether data are quantitative, qualitative, or mixed-methods (e.g. qualitative cross-sectional, quantitative experimental, mixed-methods case study).* |
| Research sample | *State the research sample (e.g. Harvard university undergraduates, villagers in rural India) and provide relevant demographic information (e.g. age, sex) and indicate whether the sample is representative. Provide a rationale for the study sample chosen. For studies involving existing datasets, please describe the dataset and source.* |

| | |
|---|---|
| Sampling strategy | *Describe the sampling procedure (e.g. random, snowball, stratified, convenience). Describe the statistical methods that were used to predetermine sample size OR if no sample-size calculation was performed, describe how sample sizes were chosen and provide a rationale for why these sample sizes are sufficient. For qualitative data, please indicate whether data saturation was considered, and what criteria were used to decide that no further sampling was needed.* |
| Data collection | *Provide details about the data collection procedure, including the instruments or devices used to record the data (e.g. pen and paper, computer, eye tracker, video or audio equipment) whether anyone was present besides the participant(s) and the researcher, and whether the researcher was blind to experimental condition and/or the study hypothesis during data collection.* |
| Timing | *Indicate the start and stop dates of data collection. If there is a gap between collection periods, state the dates for each sample cohort.* |
| Data exclusions | *If no data were excluded from the analyses, state so OR if data were excluded, provide the exact number of exclusions and the rationale behind them, indicating whether exclusion criteria were pre-established.* |
| Non-participation | *State how many participants dropped out/declined participation and the reason(s) given OR provide response rate OR state that no participants dropped out/declined participation.* |
| Randomization | *If participants were not allocated into experimental groups, state so OR describe how participants were allocated to groups, and if allocation was not random, describe how covariates were controlled.* |

# Ecological, evolutionary & environmental sciences study design

All studies must disclose on these points even when the disclosure is negative.

| | |
|---|---|
| Study description | *Briefly describe the study. For quantitative data include treatment factors and interactions, design structure (e.g. factorial, nested, hierarchical), nature and number of experimental units and replicates.* |
| Research sample | *Describe the research sample (e.g. a group of tagged Passer domesticus, all Stenocereus thurberi within Organ Pipe Cactus National Monument), and provide a rationale for the sample choice. When relevant, describe the organism taxa, source, sex, age range and any manipulations. State what population the sample is meant to represent when applicable. For studies involving existing datasets, describe the data and its source.* |
| Sampling strategy | *Note the sampling procedure. Describe the statistical methods that were used to predetermine sample size OR if no sample-size calculation was performed, describe how sample sizes were chosen and provide a rationale for why these sample sizes are sufficient.* |
| Data collection | *Describe the data collection procedure, including who recorded the data and how.* |
| Timing and spatial scale | *Indicate the start and stop dates of data collection, noting the frequency and periodicity of sampling and providing a rationale for these choices. If there is a gap between collection periods, state the dates for each sample cohort. Specify the spatial scale from which the data are taken* |
| Data exclusions | *If no data were excluded from the analyses, state so OR if data were excluded, describe the exclusions and the rationale behind them, indicating whether exclusion criteria were pre-established.* |
| Reproducibility | *Describe the measures taken to verify the reproducibility of experimental findings. For each experiment, note whether any attempts to repeat the experiment failed OR state that all attempts to repeat the experiment were successful.* |
| Randomization | *Describe how samples/organisms/participants were allocated into groups. If allocation was not random, describe how covariates were controlled. If this is not relevant to your study, explain why.* |
| Blinding | *Describe the extent of blinding used during data acquisition and analysis. If blinding was not possible, describe why OR explain why blinding was not relevant to your study.* |

Did the study involve field work? ☐ Yes ☐ No

## Field work, collection and transport

| | |
|---|---|
| Field conditions | *Describe the study conditions for field work, providing relevant parameters (e.g. temperature, rainfall).* |
| Location | *State the location of the sampling or experiment, providing relevant parameters (e.g. latitude and longitude, elevation, water depth).* |
| Access & import/export | *Describe the efforts you have made to access habitats and to collect and import/export your samples in a responsible manner and in compliance with local, national and international laws, noting any permits that were obtained (give the name of the issuing authority, the date of issue, and any identifying information).* |
| Disturbance | *Describe any disturbance caused by the study and how it was minimized.* |

# Reporting for specific materials, systems and methods

We require information from authors about some types of materials, experimental systems and methods used in many studies. Here, indicate whether each material, system or method listed is relevant to your study. If you are not sure if a list item applies to your research, read the appropriate section before selecting a response.

## Materials & experimental systems

| n/a | Involved in the study |
|---|---|
| | x Antibodies |
| | x Eukaryotic cell lines |
| x | Palaeontology and archaeology |
| | x Animals and other organisms |
| x | Clinical data |
| x | Dual use research of concern |
| x | Plants |

## Methods

| n/a | Involved in the study |
|---|---|
| x | ChIP-seq |
| | x Flow cytometry |
| x | MRI-based neuroimaging |

## Antibodies

| | |
|---|---|
| Antibodies used | The following antibodies were used :<br>anti-LAMP1 (rat, BD Pharmingen, 553792)<br>anti-LAMP1 (rabbit, Abcam, ab24170)<br>anti-LAMP2 (mouse, Abcam, ab25631)<br>anti-Rab4 (rabbit, BD Biosciences, 610889)<br>anti-Rab5 (mouse, Synaptic Systems, 108011)<br>anti-Rab5 (rabbit, Abcam, ab218624)<br>anti-Rab7 (rabbit, Abcam, ab137029)<br>anti-Rab11 (rabbit, Zymed/Invitrogen, 71-5300)<br>anti-TfR (mouse, Thermo Fisher, 13-6800)<br>anti-Tmem9 (rabbit, Proteintech, 24331-1-AP)<br>anti-Tmem9 "T9AC2" (Rabbit or guinea pig, Custom made)<br>anti-Tmem9 "T9ACt" (rabbit, Custom made)<br>anti-Tmem9b "T9BC1" (Rabbit, Custom made)<br>anti-Tmem9b "T9BC2" (Rabbit, Custom made)<br>anti-Tmem9b "T9BCt" (rabbit, Custom made)<br>anti-ClC-3 (rabbit, Custom made)<br>anti-ClC-4 (rabbit, Custom made)<br>anti-ClC-5 (rabbit or guinea pig, Custom made)<br>anti-ClC-7 (rabbit, Custom made)<br>anti-ClC-7 (rabbit, Merck, HPA043019)<br>anti-actin (mouse, Sigma, A2228)<br>anti-GFP (chicken, Aves lab, GFP-1020)<br>anti-GFP (mouse, Roche, 11814460001)<br>anti-GFP (mouse, Sigma , T5168)<br>anti-GFP (goat, R&D Systems, AF4240)<br>anti-HA (rabbit, Cell Signaling, 3724)<br>anti-HA (rat, Roche, 11867423001)<br>anti-GAPDH (mouse, Sigma, G8795)<br>anti-actin (rabbit, Sigma, A2066) |
| Validation | All commercial antibodies were validated by manufacturers (see manufacturers' web sites). Custom made, including newly generated, were validated by western blot and immunocytochemistry using HeLa knockout cells or tissue from respective knockout mice. See manuscript methods, results and reference sections in the manuscript). |

## Eukaryotic cell lines

Policy information about cell lines and Sex and Gender in Research

| | |
|---|---|
| Cell line source(s) | HEK293 and HeLa cells are both from the Leibniz-Institut DSMZ- Deutsche Sammlung von Mikroorganismen und Zellkulturen GmbH Germany |
| Authentication | Cells were authenticated by the Deutsche Sammlung von Mikroorganismen und Zellkulturen, Germany |
| Mycoplasma contamination | Cells were regularly tested for contamination by PCR |

Commonly misidentified lines
(See ICLAC register)

N/A

## Palaeontology and Archaeology

Specimen provenance

*Provide provenance information for specimens and describe permits that were obtained for the work (including the name of the issuing authority, the date of issue, and any identifying information). Permits should encompass collection and, where applicable, export.*

Specimen deposition

*Indicate where the specimens have been deposited to permit free access by other researchers.*

Dating methods

*If new dates are provided, describe how they were obtained (e.g. collection, storage, sample pretreatment and measurement), where they were obtained (i.e. lab name), the calibration program and the protocol for quality assurance OR state that no new dates are provided.*

☐ Tick this box to confirm that the raw and calibrated dates are available in the paper or in Supplementary Information.

Ethics oversight

*Identify the organization(s) that approved or provided guidance on the study protocol, OR state that no ethical approval or guidance was required and explain why not.*

Note that full information on the approval of the study protocol must also be provided in the manuscript.

## Animals and other research organisms

Policy information about studies involving animals; ARRIVE guidelines recommended for reporting animal research, and Sex and Gender in Research

Laboratory animals

Mice (Mus musculus) . Embryos 11-12.5 dpc, adults 7-22 weeks. Both genders. The following lines were used:
C57BL/6
B6;129/Sv-Clcn3tm1Tjj
B6;129-Clcn3tm3.1.1Tjj
B6;129/Sv-Clcn4tm1Tjj
B6;129/Sv-Clcn5tm1Tjj
And the newly generated knockout lines for Tmem9 and for Tmem9b described in the manuscript.
B6;tmem9 emtjj and B6;tmem9b emTjj
Xenopus laevis frogs for harvesting oocytes for electrophysiology (obtained from certified breeder)

Wild animals

N/A

Reporting on sex

N/A

Field-collected samples

N/A

Ethics oversight

All animal experiments and the generation of new animals was approved by the Berlin authorities (LAGeSo, G0111/17-21012-G0051/22; G0005/19; ZH 139; G0041/23; G0045/24; E0102/23).

Note that full information on the approval of the study protocol must also be provided in the manuscript.

## Clinical data

Policy information about clinical studies
All manuscripts should comply with the ICMJEguidelines for publication of clinical research and a completedCONSORT checklist must be included with all submissions.

Clinical trial registration

*Provide the trial registration number from ClinicalTrials.gov or an equivalent agency.*

Study protocol

*Note where the full trial protocol can be accessed OR if not available, explain why.*

Data collection

*Describe the settings and locales of data collection, noting the time periods of recruitment and data collection.*

Outcomes

*Describe how you pre-defined primary and secondary outcome measures and how you assessed these measures.*

## Dual use research of concern

Policy information about dual use research of concern

### Hazards

Could the accidental, deliberate or reckless misuse of agents or technologies generated in the work, or the application of information presented in the manuscript, pose a threat to:

| No | Yes | |
|---|---|---|
| ☐ | ☐ | Public health |
| ☐ | ☐ | National security |
| ☐ | ☐ | Crops and/or livestock |
| ☐ | ☐ | Ecosystems |
| ☐ | ☐ | Any other significant area |

## Experiments of concern

Does the work involve any of these experiments of concern:

| No | Yes | |
|---|---|---|
| ☐ | ☐ | Demonstrate how to render a vaccine ineffective |
| ☐ | ☐ | Confer resistance to therapeutically useful antibiotics or antiviral agents |
| ☐ | ☐ | Enhance the virulence of a pathogen or render a nonpathogen virulent |
| ☐ | ☐ | Increase transmissibility of a pathogen |
| ☐ | ☐ | Alter the host range of a pathogen |
| ☐ | ☐ | Enable evasion of diagnostic/detection modalities |
| ☐ | ☐ | Enable the weaponization of a biological agent or toxin |
| ☐ | ☐ | Any other potentially harmful combination of experiments and agents |

# Plants

| | |
|---|---|
| Seed stocks | N/A |
| Novel plant genotypes | N/A |
| Authentication | N/A |

# ChIP-seq

## Data deposition

☐ Confirm that both raw and final processed data have been deposited in a public database such as GEO.

☐ Confirm that you have deposited or provided access to graph files (e.g. BED files) for the called peaks.

| | |
|---|---|
| Data access links<br>*May remain private before publication.* | *For "Initial submission" or "Revised version" documents, provide reviewer access links. For your "Final submission" document, provide a link to the deposited data.* |
| Files in database submission | *Provide a list of all files available in the database submission.* |
| Genome browser session<br>(e.g. UCSC) | *Provide a link to an anonymized genome browser session for "Initial submission" and "Revised version" documents only, to enable peer review. Write "no longer applicable" for "Final submission" documents.* |

## Methodology

| | |
|---|---|
| Replicates | *Describe the experimental replicates, specifying number, type and replicate agreement.* |
| Sequencing depth | *Describe the sequencing depth for each experiment, providing the total number of reads, uniquely mapped reads, length of reads and whether they were paired- or single-end.* |
| Antibodies | *Describe the antibodies used for the ChIP-seq experiments; as applicable, provide supplier name, catalog number, clone name, and lot number.* |

| | |
|---|---|
| Peak calling parameters | *Specify the command line program and parameters used for read mapping and peak calling, including the ChIP, control and index files used.* |
| Data quality | *Describe the methods used to ensure data quality in full detail, including how many peaks are at FDR 5% and above 5-fold enrichment.* |
| Software | *Describe the software used to collect and analyze the ChIP-seq data. For custom code that has been deposited into a community repository, provide accession details.* |

# Flow Cytometry

## Plots

Confirm that:

☒ The axis labels state the marker and fluorochrome used (e.g. CD4-FITC).

☒ The axis scales are clearly visible. Include numbers along axes only for bottom left plot of group (a 'group' is an analysis of identical markers).

☒ All plots are contour plots with outliers or pseudocolor plots.

☐ A numerical value for number of cells or percentage (with statistics) is provided.

## Methodology

| | |
|---|---|
| Sample preparation | HeLa cells were seeded in 6 cm dishes and incubated at 37°C with 5% $CO_2$. After 24 hours, cells were transfected with Venus ClC-3 wild-type or mutant constructs, along with control CD4 or T9 wild type/mutants, using Lipofectamine 3000 (Invitrogen, #L3000015). Forty-eight hours post-transfection, cells were detached with 20 mM EDTA in Ringer's solution, washed (300g, 2 min, RT), and resuspended in isotonic Ringer's solution. Cells were incubated with 5 µM Lysosensor™ Yellow/Blue DND-160 (Invitrogen, #L7545) for 15 min at 37°C, washed, resuspended in 5% BSA in Ringer's solution, and analyzed by flow cytometry. |
| Instrument | LSRFortessa (BD Biosciences) |
| Software | FlowJo™ software (v10.6.1) |
| Cell population abundance | In this study we have investigated the percentage of cells having high lysosensor intensity. Primarily we applied FSC-A/ SSC-A and SSC-H/ SSC-W gating to exclude clumped, small particles and doublets. Then we selected cells, which are positive for GFP signal (FITC). And within this population of cells, we now selected cells, which have signal for lysosensor (Amcyan-A) and used for further analysis. |
| Gating strategy | For each experimental set, measurements from cells non-transfected (no signal for GFP) and non-incubated with lysosensor (no lysosensor signal) were collected and used as negative control for gating. |

☒ Tick this box to confirm that a figure exemplifying the gating strategy is provided in the Supplementary Information.

# Magnetic resonance imaging

## Experimental design

| | |
|---|---|
| Design type | *Indicate task or resting state; event-related or block design.* |
| Design specifications | *Specify the number of blocks, trials or experimental units per session and/or subject, and specify the length of each trial or block (if trials are blocked) and interval between trials.* |
| Behavioral performance measures | *State number and/or type of variables recorded (e.g. correct button press, response time) and what statistics were used to establish that the subjects were performing the task as expected (e.g. mean, range, and/or standard deviation across subjects).* |

## Acquisition

| | |
|---|---|
| Imaging type(s) | *Specify: functional, structural, diffusion, perfusion.* |
| Field strength | *Specify in Tesla* |
| Sequence & imaging parameters | *Specify the pulse sequence type (gradient echo, spin echo, etc.), imaging type (EPI, spiral, etc.), field of view, matrix size, slice thickness, orientation and TE/TR/flip angle.* |
| Area of acquisition | *State whether a whole brain scan was used OR define the area of acquisition, describing how the region was determined.* |

Diffusion MRI ☐ Used ☐ Not used

## Preprocessing

| | |
|---|---|
| Preprocessing software | *Provide detail on software version and revision number and on specific parameters (model/functions, brain extraction, segmentation, smoothing kernel size, etc.).* |
| Normalization | *If data were normalized/standardized, describe the approach(es): specify linear or non-linear and define image types used for transformation OR indicate that data were not normalized and explain rationale for lack of normalization.* |
| Normalization template | *Describe the template used for normalization/transformation, specifying subject space or group standardized space (e.g. original Talairach, MNI305, ICBM152) OR indicate that the data were not normalized.* |
| Noise and artifact removal | *Describe your procedure(s) for artifact and structured noise removal, specifying motion parameters, tissue signals and physiological signals (heart rate, respiration).* |
| Volume censoring | *Define your software and/or method and criteria for volume censoring, and state the extent of such censoring.* |

## Statistical modeling & inference

| | |
|---|---|
| Model type and settings | *Specify type (mass univariate, multivariate, RSA, predictive, etc.) and describe essential details of the model at the first and second levels (e.g. fixed, random or mixed effects; drift or auto-correlation).* |
| Effect(s) tested | *Define precise effect in terms of the task or stimulus conditions instead of psychological concepts and indicate whether ANOVA or factorial designs were used.* |

Specify type of analysis: ☐ Whole brain ☐ ROI-based ☐ Both

| | |
|---|---|
| Statistic type for inference (See Eklund et al. 2016) | *Specify voxel-wise or cluster-wise and report all relevant parameters for cluster-wise methods.* |
| Correction | *Describe the type of correction and how it is obtained for multiple comparisons (e.g. FWE, FDR, permutation or Monte Carlo).* |

## Models & analysis

| n/a | Involved in the study |
|---|---|
| ☐ | ☐ Functional and/or effective connectivity |
| ☐ | ☐ Graph analysis |
| ☐ | ☐ Multivariate modeling or predictive analysis |

| | |
|---|---|
| Functional and/or effective connectivity | *Report the measures of dependence used and the model details (e.g. Pearson correlation, partial correlation, mutual information).* |
| Graph analysis | *Report the dependent variable and connectivity measure, specifying weighted graph or binarized graph, subject- or group-level, and the global and/or node summaries used (e.g. clustering coefficient, efficiency, etc.).* |
| Multivariate modeling and predictive analysis | *Specify independent variables, features extraction and dimension reduction, model, training and evaluation metrics.* |
